# Phenotypic and genetic extended spectrum beta lactamase profiles of bacterial isolates from ICU in tertiary level hospital in Kenya

**DOI:** 10.12688/f1000research.133298.1

**Published:** 2023-05-05

**Authors:** Job Mwale, Edwin O. Magomere, Brian Maina, Leon Otieno, Frank G. Onyambu, Ali Kassim, Lucy Muchiri

**Affiliations:** 1 Department of Human Pathology, University of Nairobi, Nairobi, Nairobi County, Kenya; 2 Department of Laboratory Medicine, Meru Teaching and Referral Hospital, Meru, Meru County, Kenya; 3 Department of Laboratory Medicine, Kenyatta National Hospital, Nairobi, Nairobi County, Kenya; 4 Department of Biochemistry and Molecular Biology, Egerton University, Nakuru, Nakuru County, Kenya; 5 Centre of Microbiology, Washington State University, Nairobi, Nairobi county, Kenya; 6 Department of Clinical Medicine and Therapeutics, University of Nairobi, Nairobi, Nairobi County, Kenya; 7 Molecular Biosciences and Genomics Laboratory, Centre for Molecular Biosciences and Genomics, Nairobi, Nairobi County, Kenya; 8 Department of Medical Laboratory Sciences, Meru University, Meru, Meru county, Kenya

**Keywords:** Intensive Care Unit, anti-biotic susceptibility, gram-negative, cephalosporins, Extended Spectrum Beta Lactamase

## Abstract

**Background:** Bacterial infections in the ICUs are a threat to the lives of critically ill patients. Moreover, their vulnerable immunity predisposes them to developing bacteria-associated sepsis, further deteriorating their already fragile health. In the face of increasing drug resistance menace, the problem of bacterial infection in ICU is likely to worsen.

This study sought to assess bacterial infections in ICU setting by identifying prevalent gram-negative bacterial species and characterizing their antibiotic susceptibility patterns. Phenotypic and genetic resistance were determined among gram-negative isolates from ICU patients.

**Methods**: Cross-sectional samples collected from Kenyatta National Hospital ICU between January and June 2021 were cultured for subsequent analysis. Phenotypic identification of culture-positive samples was performed using VITEK 2 while Antibiotic susceptibility patterns were determined based on Antimicrobial Susceptibility Testing (AST) results. Cephalosporin-resistant gram-negative bacteria were assessed by PCR to detect the presence of ESBL genes.

**Results and discussion:** Out of the 168 gram-negative isolates, *Acinetobacter baumanii* was the most abundant (35%). Other isolates that were present at frequencies more than 15% are *Klebsiella pneumoniae and Escherichia. coli. A. baumaniii* is known to be a notorious bacterium in ICU due to its multidrug resistance nature. Indeed, *A. baumanii* isolates from Kenyatta National Hospital showed significantly high level of phenotypic resistance. Concordant with the high level of phenotypic resistance, we found high carriage of the ESBL genes among the isolates analysed in this study. Moreover, majority of isolates harboured all the four ESBL genes.

**Conclusion:** A high rate of phenotypic and genetic resistance was detected among the tested isolates. Resistance to cephalosporins was primarily driven by acquisition of the ESBL genes. The high-rate multidrug resistance in ICU bacterial isolates in this study has a crucial implication for ICU patient management and general antibiotics use.

## Background

The intensive care unit (ICU) is a hotspot of nosocomial infections primarily because of the extremely vulnerable population of critically ill patients, usage of invasive procedures such as catheters and ventilators ^1^^,^^2^ and immunosuppressive medication. ^3^ These infections significantly increase the burden of bacterial associated morbidity, mortality, and healthcare costs. ICU acquired infections (ICU-AI) contribute 20-25% of all nosocomial infections globally. ^4^ Recent studies have reported high risk of bloodstream infections caused by gram-negative bacteria, such as *Escherichia coli* and *Klebsiella pneumoniae* among COVID-19 patients admitted in ICU. ^5^^,^^6^

Antimicrobial resistance (AMR) is a major contributor to the problem of ICU acquired infections. AMR reduces the effectiveness of antibiotics and other antimicrobial drugs in treating these infections. Emergence of AMR leads to a higher risk of treatment failure, longer hospital stays, and increased mortality rates, as well as greater healthcare costs and resource utilization. ^7^ Drug resistant bacterial pathogens emerge and spread in the ICU environment as a result of acquisition of mutations, and selection of resistant strains, driven by indiscriminate use of antibiotics. ^8^ Additionally, gram-negative bacteria have evolved an intrinsic mechanism involving the production of extended spectrum beta lactamases (ESBLs) that breakdown the beta lactam antibiotics. ^9^ Resistance to antibiotics can be classified into: multidrug resistance (MDR), extensive drug resistance (XDR) and pan drug resistance (PDR) to reflect increasing number of antimicrobial agents affected by the resistance mechanism. ^10^ The outbreak and spread of COVID-19 also contributed to spread of drug resistant bacterial infections in ICU due to the increased number of patients requiring ICU admission. A high prevalence of bacterial pneumonia, 44% (n= 716) among covid 19 patients admitted in ICU has been reported. ^11^

Phenotypic resistance to the third generation cephalosporins (cefotaxime, ceftazidime and ceftriaxone) has been reported. ^12^^,^^13^ This resistance poses a significant public health threat since cephalosporins are valuable agents used in the management of a wide range of gram-negative infections including meningitis, Lyme disease, pseudomonas pneumonia, gram-negative sepsis, streptococcal endocarditis, melioidosis, penicillinase-producing *Neisseria gonorrhoea*, and gram-negative osteomyelitis. ^14^ Moreover, application of molecular tools to profile the ESBLs producing gram-negative bacteria have confirmed the presence of multiple ESBL genes in isolates of *Klebsiella pneumonia, Escherichia coli*, and *Proteus* species, corresponding to high-level resistance to third generation cephalosporins. ^1^

This study sought to profile phenotypic and genetic resistance to cephalosporin in bacteria isolated from ICU patients’ samples. Identification of bacterial species and phenotypic susceptibility patterns were conducted using VITEK 2 (bioMérieux). Phenotypically resistant isolates were confirmed by PCR genotyping.

## Methods

### Study design and study site

This was a cross sectional study carried out between January to June 2021 at Kenyatta National Hospital (KNH). KNH is the largest public referral and teaching hospital in Kenya with a bed capacity of approximately 1800. The hospital serves patients from the capital city with a population of over three million people. The hospital’s critical care unit department is composed of the main ICU and several other specialised units including Neurosurgery-CCU, Medical wards-CCU, Surgical ward-CCU, Neonatal-ICU, and the Casualty CCU. In this study, “ICU” to refers to both main ICU and other specialized CCUs.

### Ethical approval

This study was approved by the Kenyatta National Hospital (KNH)-University of Nairobi (UON) Ethics and research committee under the study number: P632/11/2020. Additionally, informed consent/assent were sought from participants or kin of the patient in cases of minors or unconscious patients. Written consent was obtained from next of kin for all participants but two. The two cases involved consent obtained from treating ICU physician, where the patients were incapacitated and their next of kin were unavailable to give consent. This decision was made based on the deferred consent principle backed by the following reasons

1. The research involves minimum harm to the participant
2. The deferment of consent procedure did not adversely affect the rights and welfare of the patient since the genomic testing (PCR) was carried out on the leftover bacterial isolates and not on the human DNA. These bacterial isolates are regarded as residual laboratory samples material

Patient confidentiality and data privacy was ensured by assigning unique study code to each participant. Participant metadata was collected using password protected excel data collection tool.

### Study population and sampling

Study participants included all patients admitted to various ICUs in KNH suspected to have bacterial infection during their entire period of admission. Inclusion criteria included having a gram-negative culture positive specimen. Patients with only gram-positive cultures were excluded. Sample size was determined using the Cochrane’s and Finite population correction for proportions formula. ^15^

### AST and phenotypic detection

Sample quality and quantity were reviewed prior to labelling for bacteriology assessment. Degraded samples or those with inadequate volume were excluded. Samples that passed the inclusion criteria were processed for organism identification and antimicrobial susceptibility of culture positive gram-negative isolates using the Vitek®2 ( *Biomérieux, Marcy l’Etoile, France*) with Minimum Inhibitory Concentration (MIC) breakpoints set according to CLSI 2020 guidelines. Prior to loading isolates into the VITEK® 2, bacterial suspensions were prepared by emulsifying the isolates in 0.5% saline and standardizing turbidity to 0.5 McFarland’s using a densitometer. The suspension was used for species identification, AST and phenotypic detection of ESBL producing organisms in the VITEK® 2 using gram-negative cards (GN83). Vitek®2 Advanced Expert System (AES) was used. Antimicrobial susceptibility profiles for Cefotaxime, ceftazidime and ceftriaxone were also recorded. For specimens identified phenotypically as ESBL producers, another inoculum was picked from residual specimen and stored in skimmed milk-tryptone-glucose-glycerol broth at -80°C to minimize risk of mutations during batching, awaiting PCR.

### PCR Genotyping

Isolates that showed phenotypic resistance to Cefotaxime, ceftazidime and ceftriaxone were selected and used for subsequent PCR genotyping. The Isolate II Genomic DNA kit (Bioline London, UK) was used for total DNA extraction. The kit applies affinity columns to extract genomic DNA. Proteinase K, together with cell lysis buffers containing chaotropic salt ions are used to lyse cells releasing gDNA, which is captured by the affinity resins (silica gel membrane). DNA extraction was followed according to manufacturer’s instructions and eluted in a final volume of 40 ul PCR amplification was then performed using MyTaq™ PCR mix (Bioline, London, UK) in a final volume of 20μl, comprising a master-mix, 0.4 μM of each forward and reverse primers and 3 μl of DNA template. Primers specific to ESBL encoding genes (tem, shv, ctmx and oxa) were used as described by. ^16^^,^^17^

Briefly, amplicons were analysed by gel electrophoresis run in 1% agarose gel, 1×TAE buffer and SYBR™ Safe (Invitrogen, Carlsbad, CA, USA) and a 1KB ladder at 70 volts for 30 minutes. The amplified products were visualized under Ultraviolet trans-illumination using the UVTEC Gel Documentation Systems (Cleaver Scientific, United Kingdom,) to identify presence of ESBL genes. The primer sequences and thermocycling conditions used in this study are provided in the Supplementary table 1 and Supplementary table 2 in the Data Availability section (DOI: 10.6084/m9.figshare.22369975).

### Statistical analysis

Statistical analyses were performed in MS. Excel 2010 and GraphPad Prism (version 8.0.4). Shapiro-Wilk test was used to assess data normality prior to analyses. Descriptive statistics including means and frequencies were used for data summary. Mean comparisons among three or more groups was performed using one-way ANOVA with Tukey’s post-hoc. Descriptive data was presented as mean ± SD and data considered statistically significant at p value <0.05.

## Results

### Isolate distribution

**Figure 1.**
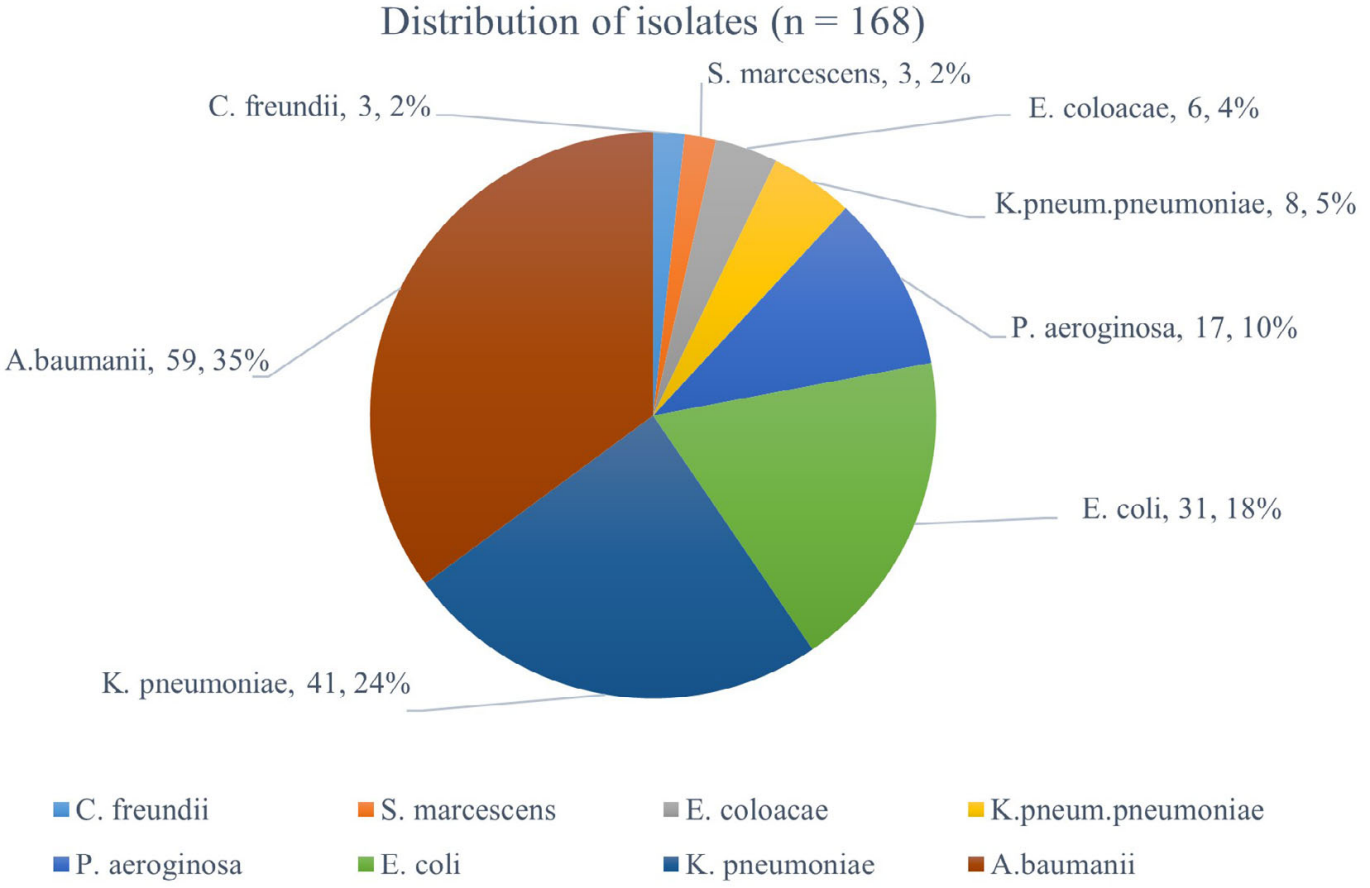
The frequency of gram-negative bacteria species identified in ICU patient samples.

A total of 168-gram-negative isolates were phenotypically identified from ICU patients’ samples. The isolates comprised of 8 gram-negative bacteria species, with *A. baumanii* being the most abundant (35%) followed by *K. pneumoniae* (24%), and *E. coli*, 18% while the remaining species were present at frequencies ≤10% ( Figure 1).

### Phenotypic susceptibility and ESB production: Majority of isolates were ESBL producers

**Figure 2.**
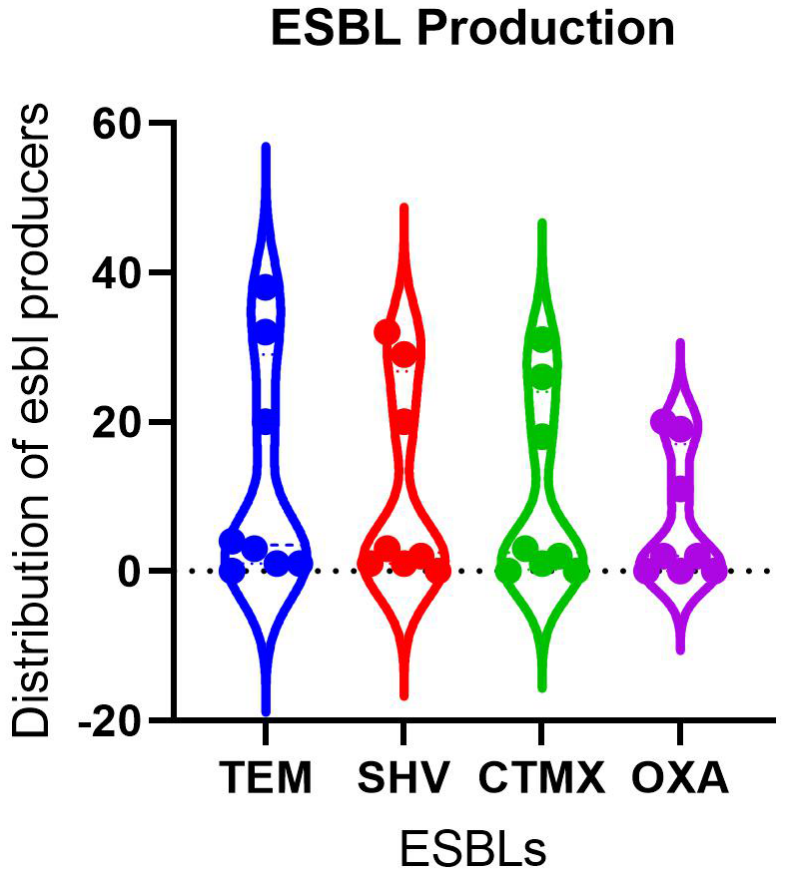
Comparison of mean number of organisms producing various ESBLs showed no statistical significance.

Phenotypic susceptibility analysis revealed high level of resistance among the bacterial isolates identified. Overall, 101/168 (60.1%) isolates were ESBL producers while 67/168 (39.9%) were ESBL non-producers. Tem was the most abundant ESBL, occurring in 99/168 followed by shv (88/168), ctmx (81/168), and oxa (54/168) ( Table 2). However, while tem was produced by most of the organisms, the differences in the differences in number of ESBL was not statistically significant ( Table 1 and Figure 2).

**Table 1.** ESBL production by gram-negative bacterial isolates from ICU patients’ specimen.

Species	tem		shv		ctxm		oxa		n	
	ESBL +ve	ESBL -ve	ESBL +ve	ESBL -ve	ESBL +ve	ESBL -ve	ESBL +ve	ESBL -ve	ESBL +ve	ESBL -ve
*C. freundii*	1	2	1	2	0	3	0	3	1	2
*S. marcescens*	1	2	1	2	1	2	0	3	1	2
*E. coloacae*	3	3	3	3	3	3	2	4	3	3
*K.pneum. pneumoniae*	0	8	0	8	0	8	0	8	0	8
*P. aeroginosa*	4	13	2	15	2	15	2	15	4	13
*E. coli*	20	11	20	11	18	13	11	20	20	11
*K. pneumoniae*	32	9	32	9	31	10	19	22	32	9
*A.baumanii*	38	2	29	11	26	14	20	20	40	19
**Total**	**99**	**50**	**88**	**61**	**81**	**68**	**54**	**95**	**101**	**67**

Comparison of various types of ESBL showed that tem was the most abundant ESBL, while oxa was the least. *A. baumani* was the most frequent ESBL producer.

### Bacterial abundance per specimen type: Tracheal aspirate specimen has the highest abundance of bacteria

The highest number of bacteria were isolated from tracheal aspirate (TA) (99/168) followed by urine (38/168) and blood (19/168) while ascitic tap, CVC tip and sputum had one isolates each. Species distribution analysis showed that *A. baumanii* were the highest in TA (38/99) Table 2.

**Table 2.** Distribution of species per specimen type.

	*A. baumanii*	*C. freundii*	*E. coloacae*	*E. coli*	*K. pneumoniae*	*K. pneumniae pneumoniae*	*P. aeruginosa*	*S. marcescens*	Total
Ascitic tap	1	0	0	0	0	0	0	0	1
Blood	9	0	3	4	3	0	0	0	19
CVC tip	0	0	0	0	0	0	1	0	1
Pus swab	0	0	0	2	2	0	3	2	9
Sputum	1	0	0	0	0	0	0	0	1
T/A	38	3	2	14	23	8	10	1	99
Urine	10	0	1	11	13	0	3	0	38
**Total**	**59**	**3**	**6**	**31**	**41**	**8**	**17**	**3**	**168**

The T/A samples had the highest number of bacterial isolates, with majority being *A. baumaii* isolates. T/A: Tracheal aspirate, CVC: central venous catheter.

### ESBL production and different parameters

Majority of patients 76% were males and the highest number of bacterial isolates were from patients aged between 21 to 40 years 75/168 and 50 out of the 75 isolates were phenotypically resistant to at one cephalosporin. Conversely, few isolates (3/168) were isolated from patients aged >80 years; all the isolates were phenotypically susceptible to all tested cephalosporins ( Table 3).

**Table 3.** Summary of ESBL production and different parameters.

		Positive (n=101)	Negative (n=67)	Total
**Age**	≤20	10	10	20
	21 – 40	50	25	75
	41 – 60	27	21	48
	61 – 80	14	8	22
	>80	0	3	3
**Gender**	Male	82	46	128
	Female	19	21	40

Comorbidities		Positive	Negative	
HIV	YES	2	0	2
	NO	99	67	166
HYPERTENSION	YES	12	1	13
	NO	89	66	155
DIABETES	YES	6	1	7
	NO	95	66	161
COVID-19	YES	7	0	7
	NO	94	67	161

Majority of patients were between 21-40 years and males were the majority. Hypertension was the most common comorbidity.

The susceptibility pattern revealed high level of phenotypic resistance against three cephalosporins (Ceftazidime, Ceftriaxone, and Cefotaxime) ( Table 4).

**Table 4.** Susceptibility patterns of various bacterial species.

	Ceftazidime			Ceftriaxone			Cefotaxime		
	S	I	R	S	I	R	S	I	R
*A. baumannii*	8	0	53	3	5	53	6	2	53
*C. freundii*	2	0	1	2	0	1	2	0	1
*E. cloacae*	4	0	4	3	0	5	3	0	5
*E. coli*	7	0	24	3	0	28	3	0	28
*K. pneumoniae*	7	6	34	4	0	43	4	0	43
*Proteus mirabilis*	0	0	1	0	0	1	0	0	1
*P. aeruginosa*	12	1	4	0	0	0	0	1	16
*S. marcescens*	2	0	1	2	0	1	2	0	1

*A. baumanii* had the highest resistance to all the three tested antibiotics followed by *K. pneumoniae* and *E. coli* respectively.

### Genotypic susceptibility

The 101 isolates that were phenotypically resistant to cephalosporin were subjected to PCR genotyping and 97 (96%) isolates harboured at least one of the four gene tested while only two (1.9%) isolates were negative for all the four genes. These results confirmed the phenotypic identification results by VITEK 2 ( Table 5).

**Table 5.** Frequency of cephalosporin resistance genes per species.

	*A. baumanii*	*E. cloacae*	*E. coli*	*K. pneumoniae*	*P. aeruginosa*
	*n=41*	*n=3*	*n=20*	*n=33*	*n=4*
CTXM	26 (63.4)	3 (100.0)	18 (90.0)	31 (93.9)	2 (50.0)
TEM	38 (92.7)	3 (100.0)	20 (100.0)	32 (97.0)	4 (100.0)
OXA	20 (48.8)	2 (66.7)	11 (55.0)	19 (57.6)	2 (50.0)
SHV	29 (70.7)	3 (100.0)	20 (100.0)	32 (97.0)	2 (50.0)

Molecular analysis detected the ESBL genes in all the bacterial species studied.

## Discussion

Bacterial infection in the ICUs represent a major burden and safety concern for patients admitted to the ICU. ^18^ Patients in ICU are often critically ill and require urgent care. As a result, they are prescribed antimicrobial therapy empirically to manage their condition while waiting for culture result. ^4^ The World Health Organization (WHO) considers this irrational use of antimicrobial in ICU a major contributor to development of antimicrobial resistance. ^19^ In light of the rampant use of antibiotics in ICU, this study was conducted to evaluate the level of bacterial colonization in various sample types drawn from ICU patients and the corresponding level of antibiotic resistant gram-negative bacteria. Additionally, susceptibility to three classes of cephalosporins (Ceftazidime, Ceftriaxone and Cefotaxime) was assessed.

*Acinetobacter baumanii*, *Klebsiela pneumoniae and E. coli* were the most abundant organisms (35%, 24%, and 18% respectively). The current study corroborates previous findings that *Acinetobacter* species (30.9%) and Klebsiella species (29.7%) followed by *Pseudomonas aeruginosa* (22.9%) were the most abundant organisms in ICU environment. ^20^ In yet another study, *Pseudomonas* species was found to be high (29.1%) in ICU setting followed by Acinetobacter (27.5%). ^21^ The trend in ICU bacterial colonization appears to be dominated by the three main organisms *Acinetobacter species*, *Klebsiella species and Pseudomonas* species, as demonstrated in previous studies ^20^^,^^21^ and corroborated by our study. Unsurprisingly, we reported *Acinetobacter baumanii* and *Klebsiela pneumoniae* as the most common organisms in ICU and resistant to all tested cephalosporins. The resistance to multiple cephalosporins might partially explain the high abundance of these bacteria in ICU. Our findings were in agreement with Saxena and colleagues’ findings that Acinetobacter and Klebsiella were resistant to multiple antibiotics. ^4^

Organism distribution varied significantly among different specimen types. Tracheal aspirate had the highest isolates (59%) followed by urine (23%) and blood (11%) while ascitic tap, CVC tip and sputum had (0.6%) each. These findings agreed with high prevalence (56%) of pulmonary colonization among ICU patients identified by tracheal aspirate culture. ^22^ Tracheal aspirate culture has been evaluated as a non-invasive method for diagnosis of ventilator-associated pneumonia colonization. ^23^ The ease of obtaining tracheal aspirate sample and availability of established protocol could explain why more tracheal aspirate samples were obtained and cultured successfully. Urine, blood and pus swabs yielded 23%, 11% and 5% of total organisms respectively. The lower proportion of culture positivity could be influenced by the small number of samples as well as the culture method.

Concordant with phenotypic susceptibility findings, we reported high level of genetic resistance in *A. baumanii, K. pneumoniae* and *E. coli. A. baumanii* is an opportunistic nosocomial pathogen that presents resistance to most antimicrobial. ^24^ This ability makes it the most persistent bacteria in ICU and has been linked to ventilator-associated pneumonia. ^25^ Carbapenem resistance in *A. baumanii* is mediated by class D β-lactamases belonging to OXA-type. Moreover, *A. baumanii* possesses an intrinsic chromosomally encoded oxacillinase *blaOXA*- *51*, which may account for the high prevalence of ^bla^OXA (48.8%) reflecting its ability to resist eradication. ^26^ We report 57.6% of *K. pneumoniae* isolates that possess OXA gene. Similar findings were recently reported demonstrating the involvement of *^bla^OXA* gene in mediating resistance to cephalosporins. ^27^

Analysis of *^bla^* CTMX, *^bla^* TEM, and *^bla^* SHV genes revealed a high resistance gene carriage in more than 50% of studied isolates. High prevalence of these markers had been reported previously in an Indonesian hospital. ^28^ Moreover, molecular surveillance of ESBL in neonates drawn from Kenya and Nigeria revealed a high prevalence of ESBL producing bacteria. ^29^ The high prevalence of ESBL producing bacteria in ICU underscore the need to heighten antibiotic resistance surveillance to provide the much-needed information to combat resistance to antibiotics.

The problem of increasing antimicrobial resistance in ICU is worrisome particularly due to the fragile nature of this category of patients. While there are no definitive measures to eradicate antibiotic resistant micro-organisms in ICUs, vaccines against these pathogens remain elusive and where available, they are unaffordable. Thus, prudent use of antibiotics in ICU to avoid widespread resistance is recommended. This study highlights the abundance of cephalosporin resistant gram-negative bacteria in ICU, which emphasized the need to heighten the fight against antibiotic resistance.

## Data Availability

Figshare. Phenotypic and genetic Extended Spectrum Beta Lactamase cephalosporin resistance profiles of bacterial isolates from ICU in Tertiary Level Hospital in Kenya. DOI: https://doi.org/10.6084/m9.figshare.22369975.v2 (Mwale, 2023). This project contains the following data: **Extended Spectrum Beta Lactamase.xlsx**: The data contain phenotypic antibiotic susceptibility values for bacterial isolates and genotypic resistance data assessed by detection of ESBL genes is also part of the data. **Raw DATA_VITEK Bacterial identification.xlsx**: Bacterial identification readings from VITEK 2. **Supplementary Materials.docx**: This file contains the PCR primer sequences and thermocycler conditions. Data are available under the terms of the Creative Commons Attribution 4.0 International license (CC-BY 4.0).
