# Supplementary material for: Phenotypic and genetic extended spectrum beta lactamase profiles of bacterial isolates from ICU in tertiary level hospital in Kenya
Source: F1000Res. 2024 Dec 10;12:469. Originally published 2023 May 5. [Version 2] doi: 10.12688/f1000research.133298.2 (PMC11754949; doi:10.12688/f1000research.133298.2)
Supplement: Peer review history

# Reviewer report: Reviewer response for version 2

**DOI:** 10.5256/f1000research.174666.r347378

**Published:** 2025-01-22

**Authors:** Tajudeen Oladunni GANIYU

**Affiliations:** 1 Fountain University, Osogbo, Osun, Nigeria

I have reviewed the corrected version of the manuscript, and the responses of the authors convince me. I recommend the acceptance of the manuscript in its current form. Thank you for allowing me to review the manuscript.

Is the work clearly and accurately presented and does it cite the current literature?

Partly

If applicable, is the statistical analysis and its interpretation appropriate?

Yes

Are all the source data underlying the results available to ensure full reproducibility?

Yes

Is the study design appropriate and is the work technically sound?

Yes

Are the conclusions drawn adequately supported by the results?

Partly

Are sufficient details of methods and analysis provided to allow replication by others?

Partly

Reviewer Expertise:

Medical Microbiology and Microbial Biotechnology

I confirm that I have read this submission and believe that I have an appropriate level of expertise to confirm that it is of an acceptable scientific standard.

# Reviewer report: Reviewer response for version 2

**DOI:** 10.5256/f1000research.174666.r347377

**Published:** 2025-01-17

**Authors:** Upendo O Kibwana

**Affiliations:** 1 Muhimbili University of Health and Allied Sciences, Dar es Salaam, Tanzania

I have reviewed the revised version and all raised comments have been addressed. I recommend approving the current version.

Is the work clearly and accurately presented and does it cite the current literature?

Yes

If applicable, is the statistical analysis and its interpretation appropriate?

Yes

Are all the source data underlying the results available to ensure full reproducibility?

Yes

Is the study design appropriate and is the work technically sound?

Yes

Are the conclusions drawn adequately supported by the results?

Yes

Are sufficient details of methods and analysis provided to allow replication by others?

Yes

Reviewer Expertise:

Bacteriology, infectious diseases

I confirm that I have read this submission and believe that I have an appropriate level of expertise to confirm that it is of an acceptable scientific standard.

# Reviewer report: Reviewer response for version 1

**DOI:** 10.5256/f1000research.146277.r316326

**Published:** 2024-10-16

**Authors:** Tajudeen Oladunni GANIYU

**Affiliations:** 1 Fountain University, Osogbo, Osun, Nigeria

The research concept is okay and relevant to the scope of the journal but the manuscript needs major revision. My comments are listed below:

1. The authors should rewrite the abstract to include a clear statement of problem and justification. The conclusion is not emphatic on what needed to be done to address the problem.
2. ESBL are more than four, what are the reasons for limiting the number of gene assessed to just four? State the four genes correctly in the abstract.
3. In the background, third paragraph and third sentence- presence of multiple ESBL genes in isolates of *Klebsiella pneumoniae, Escherichia coli *and *Proteus species*- please list the ESBL genes.
4. In the Results

- how did you differentiate ESBL producers from non ESBL producers?

 - tem should be written as TEM, shv should be SHV, ctmx should be CTX-M and oxa should be OXA.

 5. In the second paragraph of the results (Phenotypic susceptibility and ESB production-  the last sentence, - the differences in the differences in number of ESBL- delete in word in red.

 6. Discussion.

 - the discussion is poor, despite explaining and interpreting the results, the authors failed to compare their results with those of other authors. Comparing their work with those of other authors will bring out the novelty in their work and also show whether their results are better compared to earlier works.

 OTHERS

*E. coloacae* should be *Enterobacter cloacae*

 REFERENCES

- References earlier than 2019 should be updated.

Is the work clearly and accurately presented and does it cite the current literature?

Partly

If applicable, is the statistical analysis and its interpretation appropriate?

Yes

Are all the source data underlying the results available to ensure full reproducibility?

Yes

Is the study design appropriate and is the work technically sound?

Yes

Are the conclusions drawn adequately supported by the results?

Partly

Are sufficient details of methods and analysis provided to allow replication by others?

Partly

Reviewer Expertise:

Medical Microbiology and Microbial Biotechnology

I confirm that I have read this submission and believe that I have an appropriate level of expertise to confirm that it is of an acceptable scientific standard, however I have significant reservations, as outlined above.

# Response

**Published:** 2024-11-10

**Authors:** Edwin Magomere

**Affiliations:** Egerton University, Kenya

**Comment 1: **The authors should rewrite the abstract to include a clear statement of problem and justification. The conclusion is not emphatic on what needs to be done to address the problem. 

**Response**: We have rewritten the abstract to include the problem statement in the abstract, which now reads as follows: 

**Problem statement** : Bacterial infections in the Intensive Care Units are a threat to the lives of critically ill patients. Their vulnerable immunity predisposes them to developing bacteria-associated sepsis, deteriorating their already fragile health. In the face of increasing antibiotics resistance, the problem of bacterial infection in ICU is worsening.

**Justification: **Surveillance of bacterial infections in ICUs and drug resistance will help to understand the magnitude of the problem it poses and inform response strategies.

****

**Comment 2:** ESBL are more than four, what are the reasons for limiting the number of gene assessed to just four? State the four genes correctly in the abstract.

**Response:** These four ESBL genes were chosen for PCR genotyping since they were most frequently detected based on phenotypic resistance detection. Also, we restricted our study to 4 genes due to financial constraints

**Comment 3**: In the background, third paragraph and third sentence- presence of multiple ESBL genes in isolates of *Klebsiella pneumoniae, Escherichia coli *and *Proteus species*- please list the ESBL genes.

**Response**: ​We have added the various ESBL genes as recommended and included a reference to it.

**Comment 4**: How did you differentiate ESBL producers from non ESBL producers?

**Response:** We used the Phenotypic detection of extended-spectrum β-lactamase production. Specifically, we used the automated VITEK 2 ESBL test ( *Biomérieux, Marcy l’Etoile, France*).

**Description of how the method work**

 This method is based on the simultaneous assessment of the antibacterial activity of cefepime, cefotaxime and ceftazidime, measured either alone or in the presence of clavulanate. This test relies on card wells containing 1.0 mg/L of cefepime, or 0.5 mg/L of cefotaxime or ceftazidime, either alone or associated with 10 or 4 mg/L of clavulanate, respectively. After inoculation, cards are introduced into the VITEK 2 machine, and for each antibiotic tested, turbidity is measured at regular intervals. The proportional reduction of growth in wells containing a cephalosporin combined with clavulanate is then compared with that achieved by the cephalosporin alone and is interpreted as ESBL-positive or – negative through a computerized expert system. 

  We have also edited the subtitle on phenotypic detection to " **Antimicrobial Susceptibility Testing (AST) and phenotypic detection of ESBL producers**".

**Comment 5**: tem should be written as TEM, shv should be SHV, ctmx should be CTX-M and oxa should be OXA.

**Response:** We have edited the names of ESBL gene in all instances where they were not written in the right format. Thank you for drawing my attention to this.

**Comment 6**: In the second paragraph of the results (Phenotypic susceptibility and ESB production-  the last sentence, - the differences in the differences in number of ESBL- delete in word in red.

**Response**: We have deleted the words in red and re-written the sentence to read “ *^bla^* TEM was produced by most of the organisms but there were no statistically significant difference when compared to other ESBLs ”

**Comments**: Discussion.

 - the discussion is poor, despite explaining and interpreting the results, the authors failed to compare their results with those of other authors. Comparing their work with those of other authors will bring out the novelty in their work and also show whether their results are better compared to earlier works.

**Response**: We have edited the discussion to discuss our findings in context of other works in the same area and added more references. Throughout the discussion, we have endeavored to compare results to previous studies.

# Reviewer report: Reviewer response for version 1

**DOI:** 10.5256/f1000research.146277.r217591

**Published:** 2024-06-06

**Authors:** Upendo O Kibwana

**Affiliations:** 1 Muhimbili University of Health and Allied Sciences, Dar es Salaam, Tanzania

The work is relevant but needs major revision to be more understandable and reproducible

 See comments below:

 1. The authors have to be mindful of how to write scientific names properly (italicized), how to write gram stain " Gram" instead of gram, and correct all the typos. Also how the gens are written, in capital form not small letters.

 2. In the abstract the authors are talking about four genes, please indicate clearly which genes are referred to.

 3. The authors focus on ESBL genes. is OXA and ESBL gene or beta lactam gene? Please clarify and possibly change the title accordingly.

 4. In the abstract the authors are talking about high level of multidrug resistance. Can the authors clarify what  MDR is? because this is not indicated anywhere in the manuscript nor is it reflected in the results.

 5. It is not clear if this was a laboratory-based cross-sectional study or simply cross cross-sectional study. At some point, it seems the starting point was in the lab with Gram-negative isolates, while in the population section, the authors indicate they started in the ward (ICU) please indicate clearly. If the study started in the lab what was the rationale for obtaining informed consent rather than requesting a waiver for informed consent? If the study started from the ward how did you exclude pats with Gram-positive isolates prior to sample collection?

 6. The authors have to explain how they obtained isolates which subjected to identification test on VITEK. Please indicate all quality control measures observed.

 7.How many antibiotics were tested during AST? Provide the names and conc.

 8. The results section has to be rearranged to provide a better narrative. Start with where the isolates were obtained from, which isolates were isolated from where, then narrow down to the isolated ESBL.

 9. Most of the results sections can be improved to describe better what is presented in the tables. e.g. the bacterial abundance section can be improved by adding information on the isolates which were found in one type of specimen only. The ESBL   production and different production parameters section can be improved by adding information  about  other parameters; Would be good to perform a chi-square test to see if the differences between the parameters are significant or coincidental due to numbers.

 10. genotypic susceptibility section has to be described more; how many isolates carried one gene only? How many multiple? Which was the most combo of genes? In which isolates were more prominent with multiple genes etc. Also why 2 isolates were negative and not 4? 97 were positive.

 11. "The susceptibility pattern revealed high level of phenotypic resistance against three cephalosporins (Ceftazidime, Ceftriaxone, and Cefotaxime)" This sentence seems ectopic where it is placed.

 12. In the discussion section the authors should mention the settings of the studies that they make comparisons with e.g. in paragraph two.

 13. "Unsurprisingly, we reported *Acinetobacter baumanii *and *Klebsiela pneumoniae *as the most common organisms in ICU and resistant to all tested cephalosporins" This statement appears in the discussion section yet it is not indicated anywhere in your result section. Please discuss the things that are from your results.

 14. Table 1 'n' should be 'total'.

 15. Table 2 should be table 1. This is based on a comment about rearrangement.

 16. "Urine, blood and pus swabs yielded 23%, 11% and 5% of total organisms respectively. The lower proportion of culture positivity could be influenced by the small number of samples as well as the culture method." Please explain the about the culture methods referred to in this statement.

 17. Can the authors compare their genotypic results with neighbor countries and give clinical significance.

 18. I advice the authors to remove the footnotes on the tables because they are rather captions and not footnotes

 19. Table 4 is not clear. What are the numbers provided? Percentages or numbers? Provide numbers and percentages. Table not clear? What about other abx? What is the number of tested isolates for each species?

 20. The title for table 5 is not clear. What information does the table present?

Is the work clearly and accurately presented and does it cite the current literature?

Yes

If applicable, is the statistical analysis and its interpretation appropriate?

Yes

Are all the source data underlying the results available to ensure full reproducibility?

Yes

Is the study design appropriate and is the work technically sound?

Yes

Are the conclusions drawn adequately supported by the results?

Yes

Are sufficient details of methods and analysis provided to allow replication by others?

Yes

Reviewer Expertise:

Bacteriology, infectious diseases

I confirm that I have read this submission and believe that I have an appropriate level of expertise to confirm that it is of an acceptable scientific standard, however I have significant reservations, as outlined above.

# Response

**Published:** 2024-11-11

**Authors:** Edwin Magomere

**Affiliations:** Egerton University, Kenya

**Reviewer 1 comments**

**Comment 1**: The authors have to be mindful of how to write scientific names properly (italicized), how to write gram stain " Gram" instead of gram, and correct all the typos. Also how the genes are written, in capital form not small letters.

**Response:** All the typos have been corrected throughout the manuscript  and gene names written in the right format

 Typos: “gram stain” corrected to “Gram stain”,

 Italicized all scientific names

 Specific gene names have been added to the abstract: *^bla^* TEM, *^bla^* CTX-M, *^bla^* SHV and *^bla^* OXA.

**Comment 2**: In the abstract the authors are talking about four genes, please indicate clearly which genes are referred to.

**Response:** the genes we are referring to include: *^bla^* TEM, *^bla^* CTX-M, *^bla^* SHV and *^bla^* OXA. These have been added to the abstract.

**Comment 3**: The authors focus on ESBL genes. is OXA and ESBL gene or beta lactam gene? Please clarify and possibly change the title accordingly.

**Response:** OXA is an ESBL gene. Other ESBL genes that we studied include: ( *^bla^* CTX-M, *^bla^* SHV, *^bla^* TEM). We focused on four ESBL genes.

**Response: 4**. In the abstract the authors are talking about high level of multidrug resistance. Can the authors clarify what  MDR is? because this is not indicated anywhere in the manuscript nor is it reflected in the results.

**Response: **We have added the definition of MDR: Lines in the background section. The last sentence of conclusion under abstract has been rephrased to read as: “The high prevalence rate of ESBL genes  in ICU bacterial isolates shown in this study has a important implication for ICU patient management and general antibiotics use”.

**Comment 5**: It is not clear if this was a laboratory-based cross-sectional study or simply cross cross-sectional study. At some point, it seems the starting point was in the lab with Gram-negative isolates, while in the population section, the authors indicate they started in the ward (ICU) please indicate clearly. If the study started in the lab what was the rationale for obtaining informed consent rather than requesting a waiver for informed consent? If the study started from the ward how did you exclude pats with Gram-positive isolates prior to sample collection?

**Response:** The starting point of the study was the laboratory. However, we used patient samples that had been collected from ICU patients for clinical purposes. Since the samples were not originally collected for research purposes, KNH-UON Ethical review committee tasked us to obtain consent before samples could be used for research purposes. We also needed patient consent to allow us collect patient information such as co-morbidities.

 We used differential media to allow only Gram-negative bacteria to grow. Thus, we used only Gram- negative cultures for our downstream experiments.

**Comment 6:** The authors have to explain how they obtained isolates which subjected to identification test on VITEK. Please indicate all quality control measures observed.

**Response:** Samples brought to the laboratory requested by the ICU clinical team were cultured and subjected to Gram stain. Only Gram-negative isolates were analyzed on VITEK. We used commercially acquired Gram negative isolates as positive controls for each run on Vitek. For negative controls we used bacterial suspension media (saline).

**Comment 7**: How many antibiotics were tested during AST? Provide the names and conc.

**Response**: Antibiotics tested in this study include Cefotaxime, ceftazidime and ceftriaxone. The list of tested antibiotics has been added to the manuscript. The Minimum Inhibitory Concentrations (MICs) were set according to CLSI 2020 guidelines.

**Comment 8**: The results section has to be rearranged to provide a better narrative. Start with where the isolates were obtained from, which isolates were isolated from where, then narrow down to the isolated ESBL.

**Response**: The results have been rearranged to start with various sources of isolates as advised

**Comment 9**: Most of the results sections can be improved to describe better what is presented in the tables. e.g. the bacterial abundance section can be improved by adding information on the isolates which were found in one type of specimen only. The ESBL   production and different production parameters section can be improved by adding information about other parameters; Would be good to perform a chi-square test to see if the differences between the parameters are significant or coincidental due to numbers.

**Response 1:** we have added more information about species identified in specimen types with the highest number of species including the following:

 T/A harboured all the isolated tested, with a total of 99 isolates. The distribution of species in T/A showed that *A. baumanii* were the highest in TA (38/99). Urine specimen had the second highest number of species (38/168). Out of the 8 species identified, urine had 5 species, with *K.  pneumoniae *being the most frequent, identified 13 times.

**Response 2:** Table 3 has been replaced to show the outcome of Chi-Square analysis to show differences in various parameters

**Comment 10**: genotypic susceptibility section has to be described more; how many isolates carried one gene only? How many multiple? Which was the most combo of genes? In which isolates were more prominent with multiple genes etc. Also why 2 isolates were negative and not 4? 97 were positive.

**Response: Added information on**

 . ***^bla^*** TEM was the most predominant gene at **96%** (97/101), followed by SHV = 85.6% (86/101), CTX-M = 78.8% (80/101) and OXA= 52.9% (54/101). *^bla^* TEM */ ^bla^* SHV */ ^bla^* CTX-M */ ^bla^* OXA and *^bla^* TEM */ ^bla^* SHV */ ^bla^* CTX-M gene combinations were present at **49%** (n=49/101) and **25.7% **(n=26/101) respectively. Other common gene combinations included *^bla^TEM/SHV* at 6.7% (n=7/101), *^bla^TEM/CTX-M/OXA* at 1.9% (n=2/101), *^bla^TEM/SHV/OXA *at 1.9% (n= 2/101), *^bla^SHV/CTX-M* at 1.9% (n= 2/101).

**Comment 11:**  "The susceptibility pattern revealed high level of phenotypic resistance against three cephalosporins (Ceftazidime, Ceftriaxone, and Cefotaxime)" This sentence seems ectopic where it is placed.

**Response:** This sentence has been deleted as advised

**Comment 12:** In the discussion section the authors should mention the settings of the studies that they make comparisons with e.g. in paragraph two.

**Response**: The settings of previous studies have now been mentioned in the discussion section

**Comment 13:** "Unsurprisingly, we reported *Acinetobacter baumanii *and *Klebsiela pneumoniae *as the most common organisms in ICU and resistant to all tested cephalosporins" This statement appears in the discussion section yet it is not indicated anywhere in your result section. Please discuss the things that are from your results.

**Response**: This sentence has been deleted

**Comment 14:** Table 1 'n' should be 'total'.

**Response:** “n” has been edited to “Total”

**Comment 15:** Table 2 should be table 1. This is based on a comment about rearrangement.

**Response**: Tables have been rearranged accordingly

**Comment 16:** "Urine, blood and pus swabs yielded 23%, 11% and 5% of total organisms respectively. The lower proportion of culture positivity could be influenced by the small number of samples as well as the culture method." Please explain the about the culture methods referred to in this statement.

**Comment 17**: Can the authors compare their genotypic results with neighbor countries and give clinical significance.

**Response**: Results have been compared to those obtained from neighboring countries (Tanzania and Uganda) and Clinical significance highlighted **.**

**Comment 18**: I advice the authors to remove the footnotes on the tables because they are rather captions and not footnotes

**Response:** Footnotes have been removed from all tables as advised

**Comment 19**: Table 4 is not clear. What are the numbers provided? Percentages or numbers? Provide numbers and percentages. Table not clear? What about other abx? What is the number of tested isolates for each species?

**Response**: Number provided in the table is number of isolates in the categories of **S**-susceptible, **I**-intermediate and **R**-resistant. This has been clarified in the revised manuscript.

**Comment 20**: The title for table 5 is not clear. What information does the table present?

**Response:** **The title has been edited to read as “**The isolates that had resistance to all tested cephalosporins and the frequency of resistance genes”.
